# Similar rate of return to sports activity between posterior-stabilised and cruciate-retaining primary total knee arthroplasty in young and active patient

**DOI:** 10.1007/s00167-022-07176-z

**Published:** 2022-09-29

**Authors:** Riccardo D’Ambrosi, Laura Mangiavini, Rafael Loucas, Marios Loucas, Angela Brivio, Ilaria Mariani, Nicola Ursino, Filippo Migliorini

**Affiliations:** 1 IRCCS Istituto Ortopedico Galeazzi, Milan, Italy; 2 Department of Biomedical Sciences for Health, University of Milan, Milan, Italy; 3 Department of Orthopedics, Balgrist University Hospital, University of Zurich, Forchstrasse 340, 8008 Zurich, Switzerland; 4 Istituto Clinico Città Studi, Milan, Italy; 5 Royal College of Surgeons of England, London, UK; 6 Institute for Maternal, Child Health IRCCS Burlo Garofolo, Trieste, Italy; 7 Department of Orthopaedic, Trauma, and Reconstructive Surgery, RWTH University Hospital, 52074 Aachen, Germany

**Keywords:** Knee, Arthroplasty, Cruciate-retaining, Posterior-stabilised, Sport, Activity

## Abstract

**Purpose:**

Cruciate-retaining and posterior-stabilised implant designs are available for primary total knee arthroplasty. However, whether the implant design is associated with a difference in the level of activity still remains unclear. This clinical trial compared posterior-stabilised and cruciate-retaining implants in sport-related patient-reported outcome measures, range of motion, rate of return to sport, and weekly time dedicated to sport in active adults. It was also hypothesised that in young and active patients both implants lead to a similar rate of return to sport in terms of hours per week, type of sport, and joint mobility.

**Methods:**

All patients were evaluated preoperatively and for a minimum of 36 months follow-up. The University of California Los Angeles activity scores, High-Activity Arthroplasty Score, and Visual Analogue Scale were administered preoperatively and at the last follow-up. The range of motion was investigated at admission and the last follow-up. Data concerning the hours per week dedicated to sports and the type of sport practiced were also collected at admission and at the last follow-up. The Kaplan–Meier Curve was performed to compare implant survivorship.

**Results:**

Data from 227 procedures (cruciate-retaining: 109, posterior-stabilised: 118) were prospectively collected. At the last follow-up, no difference was reported in The University of California Los Angeles activity scores (*p* = 0.6), High-Activity Arthroplasty Score (*p* = 0.1), Visual Analogue Scale (*p* = 0.9), flexion (*p* = 0.7) and extension (*p* = 0.4). No difference was found in the rate of return (*p* = 0.1) and weekly hours dedicated to sport (*p* = 0.3). The Kaplan–Meier curve evidenced no statistically significant difference in implant survivorship (*p* = 0.6).

**Conclusions:**

At approximately five years of follow-up, no difference was reported between cruciate-retaining and posterior-stabilised implants in active adults in sport-related patient-reported outcomes measures, range of motion, pain, weekly time dedicated to sport, rate of return to sport, and implant survivorship.

**Level of evidence:**

Level II, prospective study.

**Supplementary Information:**

The online version contains supplementary material available at 10.1007/s00167-022-07176-z.

## Introduction

Total knee arthroplasty (TKA) aims to restore the quality of life and the activity level of patients with end-stage knee osteoarthritis [4, 24]. TKA also allows patients to return to sport at a higher level [14, 23]. Approximately 70% of patients, following primary TKA, resume their sport activities within one year postoperatively [21]. Younger and male patients reported the highest gain in physical activity following primary TKA [8].

Cruciate-retaining (CR) and posterior-stabilised (PS) implant designs are two modalities of primary TKA. CR implants are indicated in patients with stable posterior cruciate ligament (PCL); in patients with insufficient PCL, PS design is advocated. Previous clinical investigations and meta-analyses compared the PS and CR implants, agreeing that both implants promote similar outcomes, survivorship, and complication rate [26, 33]. From a biomechanical perspective, the PS implants, by removing the PCL and presenting the central prominence, create a posterior translation of the femur on the tibial plateau during flexion, increasing the rollback and the mediolateral stability [18]. On the other side, CR implants produce a paradoxical tendency for the femur to slip anteriorly during flexion [40]. However, whether this difference exerts an influence on the active population and whether the implant design is associated with a difference in the level of activity have not been previously investigated in a clinical setting.

The purpose of the current clinical trial was to compare the PS and CR implants in sport-related patient-reported outcome measures (PROMs), range of motion (ROM), weekly time dedicated to sport, and rate of return to sport in active adults. It was hypothesised that the PS implant might promote a similar time to return to sport but a greater level of activity compared to the CR design. It was also hypothesised that in young and active patients both implants lead to a similar rate of return to sport in terms of hours per week, type of sport, and joint mobility.

## Material and methods

### Study design

The present study was conducted following the principles of the Declaration of Helsinki and was approved by the ethical committee. It followed the Strengthening the Reporting of Observational Studies in Epidemiology (STROBE) statement [39]. It was prospectively registered in the Research Registry (ID: researchregistry7983). Written consent was obtained from all the enrolled participants. The 227 patients who underwent TKA during the period 10^th^ of January 2015 to 24^th^ of April 2019 were prospectively recruited at the I.R.C.C.S. Istituto Ortopedico Galeazzi, Orthopedic Surgery Department (Centro di Chirurgia Articolare Sostitutiva e Chirurgia Ortopedica, C.A.S.C.O.), Milan, Italy.

### Eligibility criteria

The inclusion criteria were: (1) knee osteoarthritis grade III to IV according to the Kellgren-Lawrence classification [19]; (2) patients younger than 65 years at the time of surgery; (3) sport-active patients regardless of type and league (4) varus-valgus deformity ≤ 5°; (5) minimum of 36 months follow-up; (6) patients able to understand the nature of the treatment and the study. The exclusion criteria were: (1) any concomitant medical condition which can exert an influence on the outcome (e.g., diabetic neuropathy, multiple sclerosis, and lateral amyotrophic sclerosis); (2) any previous surgical intervention to the affected knee (except arthroscopically partial meniscectomy); (3) severe osteoarthritis of the contralateral knee which may impair the postoperative activity level; (4) severe arthritis of the patella requiring resurfacing (5) inactive patients.

### Allocation and procedures

All patients underwent weight-bearing knee radiographies and magnetic resonance imaging (MRI) preoperatively. Patients were allocated to receive PS or CR implants. The allocation followed the anatomopathological features of the PCL based on imaging, clinical, and intra-operative findings. If the PCL was intact and effective, a CR was implanted; in case of lack of or deficient PCL, a PS was implanted. A standard medial para-patellar approach was used in all patients. No tourniquet was used. All patients received a cemented Vanguard® Knee System (Zimmer Biomet, Warsaw, IN, USA) following manufacturer instructions. Both tibial and femoral components were cemented using Refobacin® Bone Cement R (Zimmer Biomet, Warsaw, IN, USA). No augmentation or stem extension was used. No patellar resurfacing was performed. One closed suction subcutaneous drain was used and removed on the first postoperative day. The postoperative rehabilitation protocol was identical for both groups and followed published guidelines [16].

### Outcomes of interest

The clinical assessments were performed preoperatively and for a minimum of 36 months of follow-ups by two independent assessors who were not involved in the clinical management of the patients. The following sport-related PROMs were administered preoperatively and at the last follow-up: University of California Los Angeles (UCLA) activity scores [7] and High-Activity Arthroplasty Score (HAAS) [29]. Pain was evaluated at admission and at the last follow-up using the Visual Analogue Scale (VAS) [15]. The UCLA score is a clinician-based PROM to classify the activity level in one out of 10 levels, which has been validated to evaluate sport activity following joint arthroplasty [36]. The HAAS is a validated four-item PROM covering four domains (walking, running, stair climbing, and general activities). This score has been developed and validated to evaluate patients following lower limb arthroplasty, with a possible score ranging from 0 to 18 points [34].

The ROM (flexion and extension) was evaluated preoperatively and at the last follow-up using a standard long arm goniometer (Baseline Plastic Goniometers, Fabrication Enterprises Inc.) as reported by Hancock et al. [13]. ROM was measured twice following a cycle of joint flexion and extension. Data concerning the hours per week dedicated to sports and the type of sport practiced were also collected at baseline and at the last follow-up. Data concerning the following complications were recorded at the last follow-up: revisions, periprosthetic joint infection (PJI), periprosthetic fracture, and aseptic loosening. Revision was defined as any complication which led to re-operation. Implant survivorship was the time from implantation to revision. PJI was diagnosed according to the New Definition for Periprosthetic Joint Infection: From the Workgroup of the Musculoskeletal Infection Society [30]. Periprosthetic fracture was defined as fractures of the femur or tibia occurring within 15 cm from the joint line [9, 12].

### Sample size evaluation

An estimated sample of 216 subjects, 108 for each group, was required to compare UCLA activity scores between groups with a two-sided Wilcoxon–Mann–Whitney test, assuming a mean difference of 0.5, a standard deviation of 1.3 for both groups, 5% alpha, and 80% power. Given the same parameters, this sample also had 97% power to detect a pre-post difference within each group using a Wilcoxon signed-rank test.

### Patient recruitment

**Fig. 1:**
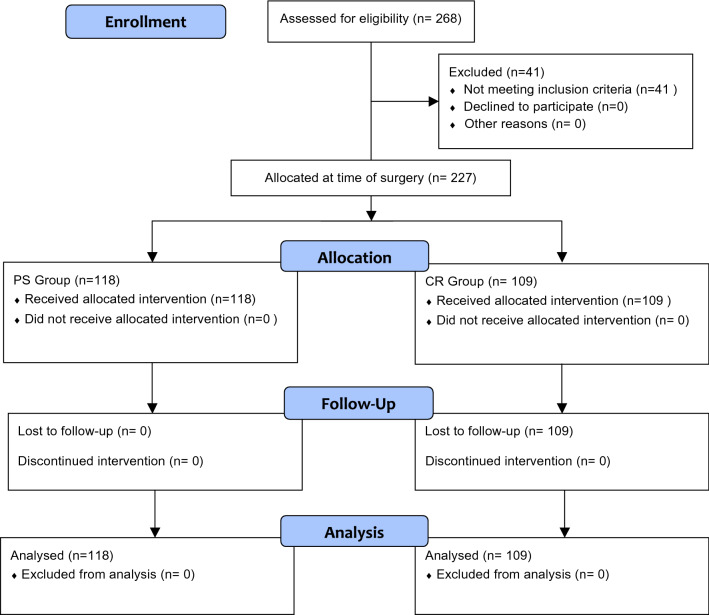
The flow diagram of the study

A total of 268 patients were initially screened. Of them, 41 were not eligible for the following reasons: severe osteoarthritis of the contralateral knee (*N* = 12), severe arthritis of the patella requiring resurfacing (*N* = 10), previous anterior cruciate ligament reconstruction (*N* = 5), previous high tibial osteotomy (*N* = 5), previous femoral/tibial fracture (*N* = 7), peripheral diabetic neuropathy (*N* = 2). A total of 227 patients were included in the present study: 118 allocated to PS and 109 to CR (Fig. 1) [1]. No patients were lost at the follow-up stage.

### Statistical analysis

Statistical analyses were conducted in R (version 4.1.1, R Development Core Team)*.* Mean and standard deviation, absolute frequencies, and percentages were used for representative statistics. The Shapiro–Wilk test was performed to investigate the distribution of continuous variables. *T*-test for normally distributed data or a Mann–Whitney *U* test for non-parametric data were performed for continuous variables, and the chi-squared test ($\chi$ ^2^) test was used to assess categorical variables*.* Implant survivorship was compared using the Kaplan–Meier Curve. Values of *P* < 0.05 were considered statistically significant. Subgroup analyses were conducted to investigate whether between implants difference exists in age (older and younger than 60 years old), sex, PROMs, ROM, and weekly hours dedicated to sport.

## Results

### Patient demographic

Between-group comparability at baseline was found in age, gender, side, surgical duration, length of the follow-up, PROMs, ROM, and weekly hours dedicated to sport. Baseline demographic and comparability is shown in Table 1.

**Table 1:** Baseline comparability

Endpoint	CR	PS	*P*-value
	(*N* = 109)	(*N* = 118)	
Age	59.54 ± 4.7	60.27 ± 4.6	0.2
Women	78 (71.6%)	72 (61.0%)	0.1
Side (right)	63 (57.8%)	69 (58.5%)	0.99
Surgical time *(*min*)*	55.7 ± 15.1	58.6 ± 15.3	0.2
Follow-up *(*months*)*	62.1 ± 18.5	65.7 ± 19.3	0.2
VAS	7.55 ± 1.2	7.11 ± 1.3	0.07
UCLA	4.71 ± 0.9	4.77 ± 1.0	0.6
HAAS	6.27 ± 1.1	6.30 ± 1.0	0.8
Extension	3.85 ± 2.7	4.48 ± 2.9	0.1
Flexion	91.99 ± 9.7	89.9 ± 10.6	0.1
Time dedicated to sport	2.34 ± 1.2	2.5 ± 1.2	0.5

*CR* cruciate retaining, *PS* posterior stabilized, *UCLA* University of California Los Angeles, *HAAS* High-Activity Arthroplasty Score, *VAS* Visual Analogue Scale

### Results syntheses

No statistically significant difference was reported in PROMs, ROM, and weekly hours dedicated to sport. These results are shown in greater detail in Table 2.

**Table 2:** Clinical comparison between the two groups at the last follow-up

Endpoint	CR (*N* = 105)	PS (*N* = 115)	*P*-value
VAS	1.5 ± 1.2	1.5 ± 1.4	0.9
UCLA	6.9 ± 1.1	7.0 ± 0.9	0.6
HAAS	10.6 ± 1.5	10.9 ± 1.4	0.1
Extension	0.2 ± 0.7	0.3 ± 0.9	0.4
Flexion	118.7 ± 5.7	118.4 ± 5.5	0.7
Weekly hours dedicated to sport	3.6 ± 1.1	3.8 ± 1.1	0.3

*CR* cruciate retaining, *PS* posterior stabilized, *UCLA* University of California Los Angeles, *HAAS* high-activity arthroplasty score, *VAS* visual analogue scale

**Fig. 2:**
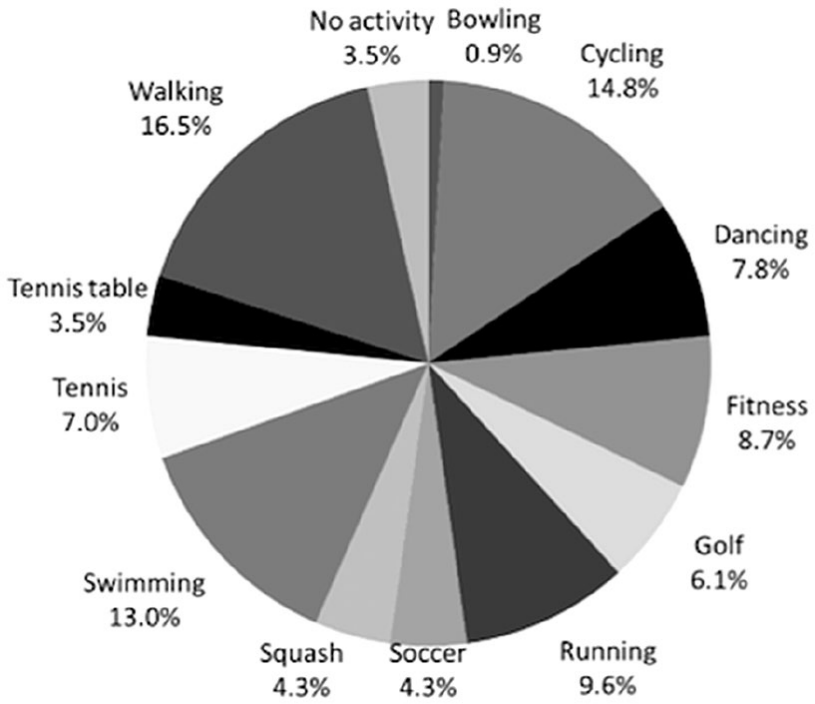
Sport activity of the patients allocated to CR at last follow-up

**Fig. 3:**
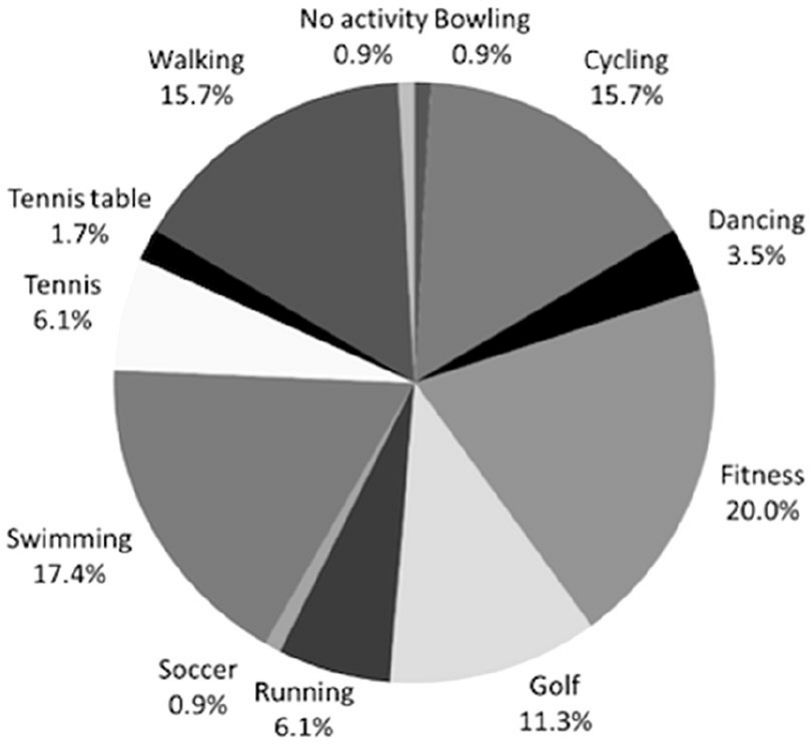
Sport activity of the patients allocated to PS at last follow-up

At baseline, all patients allocated to CR practiced sports, with a preference for swimming (14.3%) and walking (15.2%). At the last follow-up, 98.1% of the patients practiced sport, with a predominance in fitness (18.1%), walking (14.3%), and swimming (14.3%). At baseline, all patients allocated to PS practiced sports, with a preference for walking (16.5%) and cycling (14.8%). At the last follow-up, 99.1% of patients practiced sports, with a predominance in fitness (20%), swimming (17.4%), and walking (15.7%). No difference was found in the postoperative rate of return to sport between CR and PS (*p* = 0.1). Details of sports practiced are reported in Figs. 2, 3.

### Complications and survivorship

**Fig. 4:**
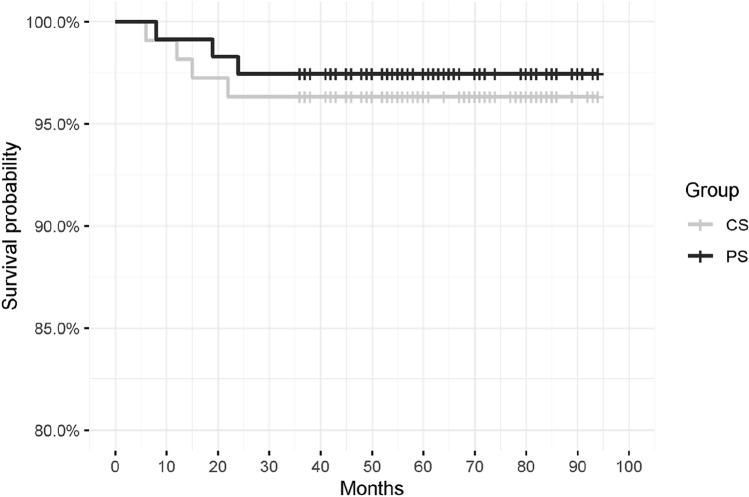
Kaplan–Meier curve

In the CR group, there were 4 revisions (3.7%): one periprosthetic fracture at 22 months postoperatively, aseptic loosening in two patients at 11 and 15 months postoperatively, and one infection at 6 months postoperatively. In the PS group, there were 3 revisions (2.5%): aseptic loosening in two patients at 6 and 8 months postoperatively and one infection at 19 months postoperatively. The Kaplan–Meier curve (Fig. 4) evidenced no statistically significant difference in implant survivorship (*p* = 0.6).

## Discussion

The most important finding of the present study was that no difference was reported between CR and PS implants in activity PROMs, ROM, weekly hours dedicated to sport, and the rate of return to sport at approximately 5 years of follow-up in active adults. The same endpoints demonstrated no difference between younger and older patients or men and women. No statistically significant difference in implant survivorship according to the Kaplan–Meier curve was observed.

The PCL is a two-bundle ligament which dynamically stabilises the knee during flexion and extension and, given its mechanoreceptors, is involved in the transmission of body kinaesthesia and proprioception [27, 28]. However, in elderlies the PCL is often unstable, degenerate, frayed, partially or totally broken and a PS implant should be used [2]. From a biomechanical perspective, the PS implants, by removing the PCL and presenting the central prominence, generate a posterior translation of the femur to the tibial plateau during flexion, increasing the rollback and the mediolateral joint stability [17]. On the other hand, CR implants generate a paradoxical tendency of the femur to slip anteriorly during flexion [11]. Although these differences exist, previous meta-analyses that compared the two implants did not find any difference in the outcomes. A previous Cochrane systematic review including 17 RCTs (1810 patients, 2206 procedures) found no difference in the clinical and pain subscales of the Knee Society Scores (KSS), Western Ontario and McMaster Universities Osteoarthritis Index (WOMAC) total score, implant survivorship, and rate of revision [38]. The authors found a minimally greater functional Knee Society Score and ROM in the PS group [38]. However, the authors remarked that such minimal differences have a dubious impact on the clinical outcome [38]. Moreover, the methodological quality and the quality of reporting of the studies were highly variable. In a recent meta-analysis including 36 articles (4052 patients, 4884 procedures), a slightly greater ROM and functional Knee Society Score was evidenced in the PS group [26]. No difference was found in the other PROMs and the rate of anterior knee pain, joint instability, and revision surgery [26]. Previous clinical trials which compared the ROM between the two implants reported heterogeneous results. Most studies reported a gain in ROM of 5 to 10 degrees in favour of the PS cohort [3, 10, 37]. Few clinical investigations reported similar [5, 20, 31] or slightly greater ROM in the CR implant [22, 35]. In the present study, we were unable to show a difference in ROM between the two implants. Previous clinical trials evaluated the pain using the pain subscale of the WOMAC score, with no substantial difference between the PS and CR implants [3, 32]. The majority of previously published clinical trials focused on elderlies, without reporting information with regard to their sport activity. Although previous studies found similarities in the clinical outcome, whether differences in implant design, presence of PCL, and biomechanics between CR and PS implants have an impact on sport activity has not been previously investigated in a clinical setting. In the present study, we referred to the UCLA and HAAS scores to compare the sport activity of the two implants. Data from the present study revealed no difference in the UCLA and HAAS scores, indicating that both implants allow patients to have similar activity performance. Moreover, weekly hours dedicated to sport and the rate of return to sport were also similar between the PS and CR implants.

The present study certainly has limitations. The lack of randomised allocation increases the risk of selection bias. The lack of blinding of patients and assessors increases the risk of detection and attrition biases. However, we must point out that randomisation and blinding in elective surgery is not well accepted by patients and surgeon alike. General health measures were not considered for analysis and the investigations have been conducted irrespective of the aetiology of osteoarthritis (idiopathic, osteonecrosis, dysplasia, trauma). The patients were enrolled irrespective of the level and league of sport practiced; although this may impact the reliability of the present study, we remark that all patients performed sport at the recreational level and none at elite or competition level. At our institution we retained the patella as standard; whether patellar resurfacing affects the clinical outcome has not been fully clarified [6, 25, 41].

The study setting is a high-volume, tertiary referral hospital, so our findings may not be generalisable to institutions where TKAs are not performed as frequently. Moreover, this study followed patients for only 3 years post-surgically; however, patient activity levels may change beyond this period.

Finally, the time to return to sport was not assessed, which could have provided additional value to the results of the present investigation. These findings help to manage patient expectations. In particular, for young and active patients with knee osteoarthritis, both the CR and PS implants can be considered safe procedures which allow a high return to sports activity in the midterm.

These findings inform shared decision-making and can help to manage patient expectations after surgery. In particular, in young active patients with or without the PCL, both CR and PS implants should be considered the gold standard in total knee replacement surgery with a high level of return to sports activity.

## Conclusions

No difference was reported between the CR and PS implants in activity PROMs, ROM, weekly hours dedicated to sport, and the rate of return to sport at approximately five years of follow-up in active adults. The same endpoints demonstrated no difference between younger and older patients or men and women. No statistically significant difference in implant survivorship according to the Kaplan–Meier curve has been evidenced.

## Supplementary Information

Below is the link to the electronic supplementary material.

Supplementary file1 (XLS 82 KB)

## Data Availability

Raw data have been submitted as supplementary material to the Journal.
